# Correction: Asymmetrical diversification of the receptor-ligand interaction controlling self-incompatibility in Arabidopsis

**DOI:** 10.7554/eLife.112595

**Published:** 2026-07-09

**Authors:** Maxime Chantreau, Céline Poux, Marc F Lensink, Guillaume Brysbaert, Xavier Vekemans, Vincent Castric

**Keywords:** *A. thaliana*

Chantreau M, Poux C, Lensink MF, Brysbaert G, Vekemans X, Castric V. 2019. Asymmetrical diversification of the receptor-ligand interaction controlling self-incompatibility in Arabidopsis. *eLife* **8**:e50253. doi: 10.7554/eLife.50253.

Published 25 November 2019

We recently noticed that the published version of our article contains a duplication of the two top panels of Figure 3. The version in the original manuscript for the first round of review was correct and contained no duplication, and the duplication error was introduced when the revised manuscript was submitted for assessment due an error in the script used for visualization. The original published data remain unchanged (Figure 3—source data 1). The R script used to create the new figure has been deposited on github (https://github.com/vincentcastric/AncestralResurrection). The modification does not alter any of the conclusions and the main text thus remains unchanged.

The corrected Figure 3 (upper left panel corrected) is shown here:

**Figure 1:**
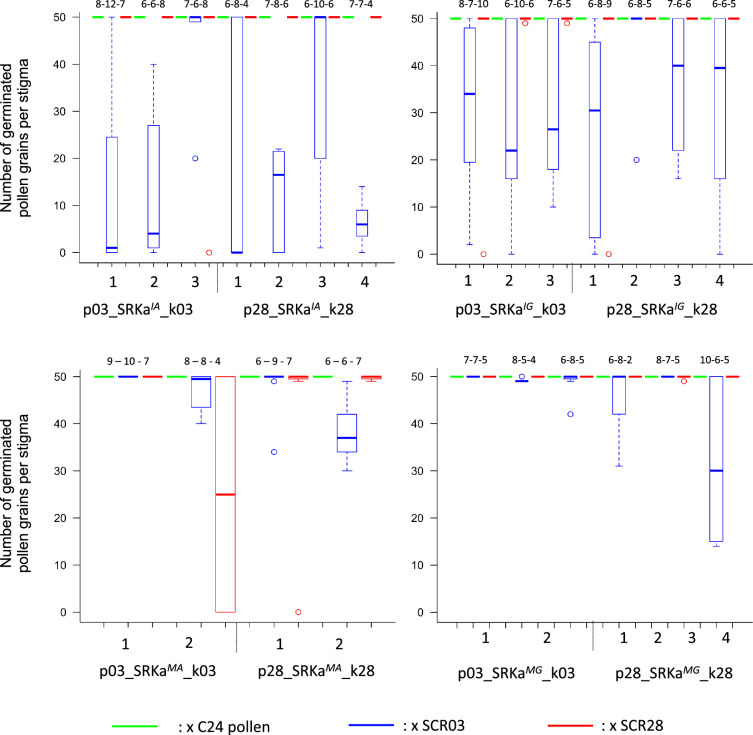


The originally published Figure 3 is shown for reference:

**Figure 2:**
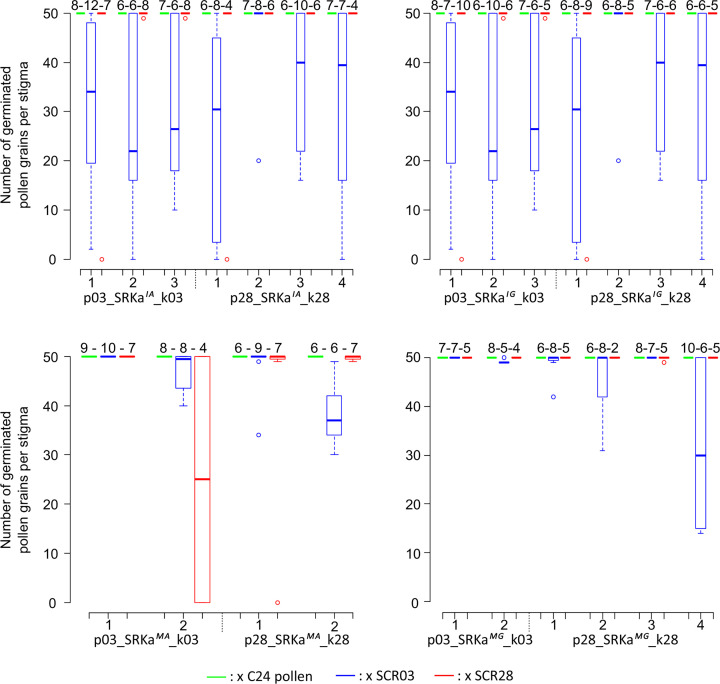


The article has been corrected accordingly.

