# Supplementary material for: Asymmetrical diversification of the receptor-ligand interaction controlling self-incompatibility in Arabidopsis
Source: eLife. 2019 Nov 25;8:e50253. doi: 10.7554/eLife.50253 (PMC6908432; doi:10.7554/eLife.50253)
Supplement: Supplementary file 1. — Following the eSRK:SCR template structure where two SCR molecules interact with two SRK proteins to form a heterotetramer (Ma et al., 2016), we indicate the two SRK molecules with their chain identifier A and B and the two SCR molecules with G and H. For each complex, the number of amino acids involved and the number of atomic contacts are defined for each protein chain interaction (AG, AH, BG and BH). Underlined numbers in the column ‘involved aa’ correspond to the number of amino acids involved in both cognate and non-cognate interactions. [file elife-50253-supp1.doc]

| SRK receptor | Chain | SCR ligand | Chain | SRK involved aa | SCR involved aa | Atomic contacts |
| --- | --- | --- | --- | --- | --- | --- |
| S03 | A | S03 | G | 16+6=22 | 9+9=18 | 464 |
|  | A |  | H | 19+13=32 | 12+7=19 | 399 |
|  | B |  | G | 23+1=24 | 7+10=17 | 273 |
|  | B |  | H | 15+2=17 | 7+9=16 | 407 |
|  |  |  |  |  |  | **1543** |
| S28 | A | S28 | G | 15+1=16 | 8+7=15 | 277 |
|  | A |  | H | 17+7=24 | 10+7=17 | 326 |
|  | B |  | G | 17+18=35 | 4+14=18 | 471 |
|  | B |  | H | 15+2=17 | 9+7=16 | 321 |
|  |  |  |  |  |  | **1395** |
| S03 | A | S28 | G | 16+5=21 | 9+9=18 | 366 |
|  | A |  | H | 19+8=27 | 10+7=17 | 389 |
|  | B |  | G | 23+4=28 | 4+13=17 | 380 |
|  | B |  | H | 15+8=23 | 9+9=18 | 411 |
|  |  |  |  |  |  | **1546** |
| S28 | A | S03 | G | 15+5=20 | 9+6=15 | 378 |
|  | A |  | H | 17+10=27 | 12+7=19 | 353 |
|  | B |  | G | 17+3=20 | 7+10=17 | 244 |
|  | B |  | H | 15+5=20 | 7+10=17 | 353 |
|  |  |  |  |  |  | **1328** |
