# Supplementary material for: Asymmetrical diversification of the receptor-ligand interaction controlling self-incompatibility in Arabidopsis
Source: eLife. 2019 Nov 25;8:e50253. doi: 10.7554/eLife.50253 (PMC6908432; doi:10.7554/eLife.50253)
Supplement: Supplementary file 2. [file elife-50253-supp2.doc]

| Sequence | Accession number |
| --- | --- |
| AhSRK03 | KJ772380.1 |
| AhSRK04 | KJ461484.1 |
| AhSRK08 | EU075130.1 |
| AhSRK09 | EU075131.1 |
| AhSRK10 | KM592810.1 |
| AhSRK19 | EU075140.1 |
| AhSRK23 | EU878008.1 |
| AhSRK27 | EU878012.1 |
| AhSRK28 | KJ461478.1 |
| AhSRK29 | KM592798.1 |
| AlSRK06 | GQ351354.1 |
| AlSRK08 | JX464638.1 |
| AlSRK14 | KJ772405.1 |
| AlSRK18 | KJ772412.1 |
| AlSRK29 | AY186776.1 |
| AlSRK39 | KJ772418.1 |
| CgrSRK1 | DQ530637.1 |
| CgrSRK4 | DQ530640.1 |
| CgrSRK5 | DQ530641.1 |
| CgrSRK6 | DQ530642.1 |
