# Supplementary material for: Asymmetrical diversification of the receptor-ligand interaction controlling self-incompatibility in Arabidopsis
Source: eLife. 2019 Nov 25;8:e50253. doi: 10.7554/eLife.50253 (PMC6908432; doi:10.7554/eLife.50253)
Supplement: Supplementary file 3. — np is the number of parameters in the model; lnL is the log likelihood score; AIC (Akaike Information criterion = −2*lnL+2*np) is a measure of the goodness of fit of an estimated statistical model; ω is the nonsynonymous/synonymous substitution ratio; LR is the likelihood ratio: df is the degree of freedom in LRT (Likelihood Ratio Test); *** Highly significant (p-value<0.0001). [file elife-50253-supp3.doc]

| **Model** | **np** | **lnL** | **AIC** | **ω** |
| --- | --- | --- | --- | --- |
| **M0** | 43 | -8976.64 | 18039 | ω_0_=0.51 |
| **M0FMutSel0** | 56 | -8949.78 | 18012 | ω_0_=0.55 |
| **M0FMutSel** | 97 | -8878.19 | 17950 | ω_0_=0.53 |
| **M3** | 47 | -8765.23 | 17624 | ω_0_=0.03, ω_1_=0.48, ω_2_=1.72  p_0_=0.30, p_1_=0.52, p_2_= 0.18 |
| **M3FMutSel0** | 60 | -8745.69 | 17607 | ω_0_=0.19, ω_1_=1.41  p_0_=0.65, p_1_=0.35 |
| **M3FMutSel** | 101 | -8684.54 | 17567 | ω_0_=0.19, ω_1_=1.34  p_0_=0.64, p_1_=0.36 |
| **LRT M3FMutSel *vs* M3FMutSel0: LR = 122.3 (df=41)***** | | | | |
