# Supplementary material for: Asymmetrical diversification of the receptor-ligand interaction controlling self-incompatibility in Arabidopsis
Source: eLife. 2019 Nov 25;8:e50253. doi: 10.7554/eLife.50253 (PMC6908432; doi:10.7554/eLife.50253)
Supplement: Supplementary file 4. [file elife-50253-supp4.doc]

| Construct | Primers | Sequences |
| --- | --- | --- |
| AhSRK03 | attB1.-1940.-F  attB2.4555.R | 5’-GGGGACAAGTTTGTACAAAAAAGCAGGCTAACCCTGGCTTACTGACTTG-3’  5’-GGGGACCACTTTGTACAAGAAAGCTGGGTAATCGCCCGGTTATTGCCTG-3’ |
| AhSRK28 | attB1.-1950.F  attB2.4555.R | 5’-GGGGACAAGTTTGTACAAAAAAGCAGGCTAGGTTAGTCCATAGCCCTTG-3’  5’-GGGGACCACTTTGTACAAGAAAGCTGGGTAATCGCCCGGTTATTGCCTG-3’ |
| AhSRK03p:GFP | attB1.-1940.F  attB2.-1.R | 5’-GGGGACAAGTTTGTACAAAAAAGCAGGCTAACCCTGGCTTACTGACTTG-3’  5’-GGGGACCACTTTGTACAAGAAAGCTGGGTCTCTCTCTCTACCACTGTGC-3’ |
| AhSRK28p:GFP | attB1.-1950.F  attB2.-3.R | 5’-GGGGACAAGTTTGTACAAAAAAGCAGGCTAGGTTAGTCCATAGCCCTTG-3’  5’-GGGGACCACTTTGTACAAGAAAGCTGGGTCTCTCTCTACCACTGTGCTC-3’ |
| p03_SRKa_k03 | attB4.-1940.F  attB1r.-1.R  -  attB2r.1393.F  attB3.4555.R | 5’-GGGGACAACTTTGTATAGAAAAGTTGAACCCTGGCTTACTGACTTG-3’  5’-GGGGACTGCTTTTTTGTACAAACTTGCTCTCTCTCTACCACTGTGC-3’  -  5’-GGGGACAGCTTTCTTGTACAAAGTGGCAACTTCCGGTTCCATATCC-3’  5’-GGGGACAACTTTGTATAATAAAGTTGAATCGCCCGGTTATTGCCTG-3’ |
| p28_SRKa_k28 | attB4.-1950.F  attB1r.-3.R  -  attB2r.1393.F  attB3.4555.R | 5’-GGGGACAACTTTGTATAGAAAAGTTGAGGTTAGTCCATAGCCCTTG-3’  5’-GGGGACTGCTTTTTTGTACAAACTTGCTCTCTCTACCACTGTGCTC-3’  -  5’-GGGGACAGCTTTCTTGTACAAAGTGGCAACTTCCGGTTCCATATCC-3’  5’-GGGGACAACTTTGTATAATAAAGTTGAATCGCCCGGTTATTGCCTG-3’ |
