# Supplementary material for: Asymmetrical diversification of the receptor-ligand interaction controlling self-incompatibility in Arabidopsis
Source: eLife. 2019 Nov 25;8:e50253. doi: 10.7554/eLife.50253 (PMC6908432; doi:10.7554/eLife.50253)
Supplement: Supplementary file 5. [file elife-50253-supp5.doc]

| **Key Resources Table** | | | | |
| --- | --- | --- | --- | --- |
| **Reagent type (species) or resource** | **Designation** | **Source or reference** | **Identifiers** | **Additional information** |
| Gene (*Arabidopsis halleri*) | AhSRK03  AhSRK04  AhSRK08  AhSRK09  AhSRK10  AhSRK19  AhSRK23  AhSRK27  AhSRK28  AhSRK29 | NCBI | KJ772380.1  KJ461484.1  EU075130.1  EU075131.1  KM592810.1  EU075140.1  EU878008.1  EU878012.1  KJ461478.1  KM592798.1 |  |
| Gene (*Arabidopsis lyrata*) | AlSRK06  AlSRK08  AlSRK14  AlSRK18  AlSRK29  AlSRK39 | NCBI | GQ351354.1  JX464638.1  KJ772405.1  KJ772412.1  AY186776.1  KJ772418.1 |  |
| Gene (*Capsella grandiflora)* | CgrSRK1  CgrSRK4  CgrSRK5  CgrSRK6 | NCBI | DQ530637.1  DQ530640.1  DQ530641.1  DQ530642.1 |  |
| gene (*Arabidopsis halleri*) | BAC sequences of A. halleri S03 | NCBI  PMID: [22457631](https://www.ncbi.nlm.nih.gov/pubmed/22457631/) | KJ772378-KJ772385 |  |
| gene (*Arabidopsis halleri*) | BAC sequences of A. halleri S28 | NCBI  PMID: [22457631](https://www.ncbi.nlm.nih.gov/pubmed/22457631/) | KJ461475-KJ461478 |  |
| strain, strain background ((E. coli, DH5α)) | Subcloning Efficiency™ DH5α Competent Cells | Thermo Fisher Scientific | 18265017 |  |
| strain, strain background (Agrobacterium tumefasciens GV2260) | Agrobacterium strain GV2260 | PMID:1966276 |  |  |
| transfected construct (*Arabidopsis thaliana*) | *AhSRK28* into pK7WG plasmid | This paper |  | Refer to Materials and methods |
| transfected construct (*Arabidopsis thaliana*) | *AhSRK28* into pK7WG plasmid | This paper |  | Refer to Materials and methods |
| transfected construct (*Arabidopsis thaliana*) | *AhSRK03p:GFP* into pKGWFS7.0 plasmid | This paper |  | Refer to Materials and methods |
| transfected construct (*Arabidopsis thaliana*) | *AhSRK28p:GFP* into pKGWFS7.0 plasmid | This paper |  | Refer to Materials and methods |
| transfected construct (*Arabidopsis thaliana*) | p03_SRKa*^IA^*_k03 into pK7m34GW plasmid | This paper |  | Refer to Materials and methods |
| transfected construct (*Arabidopsis thaliana*) | p28_SRKa*^IA^*_k28 into pK7m34GW plasmid | This paper |  | Refer to Materials and methods |
| transfected construct (*Arabidopsis thaliana*) | p03_SRKa*^IG^*_k03 into pK7m34GW plasmid | This paper |  | Refer to Materials and methods |
| transfected construct (*Arabidopsis thaliana*) | p28_SRKa*^IG^*_k28 into pK7m34GW plasmid | This paper |  | Refer to Materials and methods |
| transfected construct (*Arabidopsis thaliana*) | p03_SRKa*^MA^*_k03 into pK7m34GW plasmid | This paper |  | Refer to Materials and methods |
| transfected construct (*Arabidopsis thaliana*) | p28_SRKa*^MA^*_k28 into pK7m34GW plasmid | This paper |  | Refer to Materials and methods |
| transfected construct (*Arabidopsis thaliana*) | p03_SRKa*^MG^*_k03 into pK7m34GW plasmid | This paper |  | Refer to Materials and methods |
| transfected construct (*Arabidopsis thaliana*) | p28_SRKa*^MG^*_k28 into pK7m34GW plasmid | This paper |  | Refer to Materials and methods |
| biological sample (*Arabidopsis halleri*) | *Arabidopsis halleri* pollen with SCR03 | PMID: 22457631 |  |  |
| biological sample (*Arabidopsis halleri*) | *Arabidopsis halleri* pollen with SCR28 | PMID: 22457631 |  |  |
| recombinant DNA reagent | Synthesized putative ancestral S-domain surrounded by attL1 and attL2 sequences: SRKa*^IA^* | Thermo Fisher Scientific |  | >SRKa*^IA^* CAAATAATGATTTTATTTTGACTGATAGTGACCTGTTCGTTGCAACAAATTGATGAGCAATGCTTTTTTATAATGCCAACTTTGTACAAAAAAGCAGGCTAGATGAGAGGTGCAGTACCAAATTACCACCATTCTCACAACTTTTTCTTCTTCCTGTTTGTTGTCTCAGTTCTGTTTCGTCCTGCGTTCTCGATCTTTGCCAATACTTTGTCGTCTACGGAATCTCTGACAATCGCAAGCAACCGAACCATCGTCTCTCTCGGTGATGATTTCGAACTTGGTTTCTTCAGACCCGCTGCAAGTCTTCGAGAGGGTGATCGTTGGTATCTCGGGATTTGGTACAAGACAATCTCGGTAAGAACCTATGTATGGGTTGCCAACAGAGATCATCCTATCTCCAGTTCTGATGGAACCCTCAAAATTTCCGGAATTAACCTTGTCCTCCTCAATCAATCTAACATCACTGTCTGGTCGACGAATCTGACTGGTGCTGTGAGATCTCCAGTGGTAGCAGAGCTTCTCCCTAATGGCAATTTCGTGCTTAGAGACTCCAAAACCAACGGGCAAGATGGATTTTTGTGGCAGAGCTTTGATTACCCGACAGATACTTTACTCCCGCATATGAAATTGGGTTTGGATCTCAAAACAGGAAACAACAGATTTCTTACATCCTGGAAAAACTCATATGATCCGTCAAGCGGGTATCTTTCGTACAAACTCGAAATACAAGGGTTGCCTGAGTTTCTTATGTGGAGGAGCGGAGGGCCAGTGTTCCGGAGTGGTCCGTGGGATGGAATCCGGTTTAGTGGCATACCAGAGATGCAAAGATGGAAGTTCGTTAACATTGTTTACAATTTCACGGAGAACAAAGAGGATGTCGCCTTCACTTATAGAGTTACCACCCCCAATGTCTACGCGAAATTGACGATGAGATTCGACGGGTTTTTAGAACTATCCACATGGGATCCGGAAATGTTGGAATGGAACGTGTTCTGGGTTTCATCGACAGCCGATTGTGATATTTACATGGGGTGTACTCCTTATAGCTTCTGTGACACGAACACAACGCCAAAGTGTAACTGTATCAAAGGATTTGAGCCGAGGAACCCTCAGGGAGGAGCATTGGATAACACATCCACTGAGTGTGTAAGGAAGACGCAGTTAAATTGCAATGGAGATGGATTTTTCTGGCTGAGGAATATGAAGCTGCCGGACACATCGGGTGCTATTGTGGACAAGAGGATTGGGTTAAAGGAATGTGAGGAGAGGTGCATTGAGAATTGTAACTGTACTGCGTTTGCTAATACGAATATCCAAGATGGTGGGTCGGGTTGTGTGCTTTGGACTCGCGAGCTCGCAGATATTCGGAGATATGTCGATGCGGGTCAAGATCTTTATGTCAGATTGGCTGCTGTTGATCTCGGTTAGCTCTATCCCGTAGAATTATTACACGTAATGTATCCTGAAAATTCAGGGATCCACCCAAATTACCCAGCTTTCTTGTACAAAGTTGGCATTATAAGAAAGCATTGCTTATCAATTTGTTGCAACGAACAGGTCACTATCAGTCAAAATAAAATCATTATTTGCC-- |
| recombinant DNA reagent | Synthesized putative ancestral S-domain surrounded by attL1 and attL2 sequences: SRKa*^IG^* | Thermo Fisher Scientific |  | > SRKaIG  CAAATAATGATTTTATTTTGACTGATAGTGACCTGTTCGTTGCAACAAATTGATGAGCAATGCTTTTTTATAATGCCAACTTTGTACAAAAAAGCAGGCTAGATGAGAGGTGCAGTACCAAATTACCACCATTCTCACAACTTTTTCTTCTTCCTGTTTGTTGTCTCAGTTCTGTTTCGTCCTGCGTTCTCGATCTTTGCCAATACTTTGTCGTCTACGGAATCTCTGACAATCGCAAGCAACCGAACCATCGTCTCTCTCGGTGATGATTTCGAACTTGGTTTCTTCAGACCCGCTGCAAGTCTTCGAGAGGGTGATCGTTGGTATCTCGGGATTTGGTACAAGACAATCTCGGTAAGAACCTATGTATGGGTTGCCAACAGAGATCATCCTATCTCCAGTTCTGATGGAACCCTCAAAATTTCCGGAATTAACCTTGTCCTCCTCAATCAATCTAACATCACTGTCTGGTCGACGAATCTGACTGGTGCTGTGAGATCTCCAGTGGTAGCAGAGCTTCTCCCTAATGGCAATTTCGTGCTTAGAGACTCCAAAACCAACGGGCAAGATGGATTTTTGTGGCAGAGCTTTGATTACCCGACAGATACTTTACTCCCGCATATGAAATTGGGTTTGGATCTCAAAACAGGAAACAACAGATTTCTTACATCCTGGAAAAACTCATATGATCCGTCAAGCGGGTATCTTTCGTACAAACTCGAAATACAAGGGTTGCCTGAGTTTCTTATGTGGAGGAGCGGAGGGCCAGTGTTCCGGAGTGGTCCGTGGGATGGAATCCGGTTTAGTGGCATACCAGAGATGCAAAGATGGAAGTTCGTTAACATTGTTTACAATTTCACGGAGAACAAAGAGGATGTCGCCTTCACTTATAGAGTTACCACCCCCAATGTCTACGCGAAATTGACGATGAGATTCGACGGGTTTTTAGAACTATCCACATGGGATCCGGAAATGTTGGAATGGAACGTGTTCTGGGTTTCATCGACAGGCGATTGTGATATTTACATGGGGTGTACTCCTTATAGCTTCTGTGACACGAACACAACGCCAAAGTGTAACTGTATCAAAGGATTTGAGCCGAGGAACCCTCAGGGAGGAGCATTGGATAACACATCCACTGAGTGTGTAAGGAAGACGCAGTTAAATTGCAATGGAGATGGATTTTTCTGGCTGAGGAATATGAAGCTGCCGGACACATCGGGTGCTATTGTGGACAAGAGGATTGGGTTAAAGGAATGTGAGGAGAGGTGCATTGAGAATTGTAACTGTACTGCGTTTGCTAATACGAATATCCAAGATGGTGGGTCGGGTTGTGTGCTTTGGACTCGCGAGCTCGCAGATATTCGGAGATATGTCGATGCGGGTCAAGATCTTTATGTCAGATTGGCTGCTGTTGATCTCGGTTAGCTCTATCCCGTAGAATTATTACACGTAATGTATCCTGAAAATTCAGGGATCCACCCAAATTACCCAGCTTTCTTGTACAAAGTTGGCATTATAAGAAAGCATTGCTTATCAATTTGTTGCAACGAACAGGTCACTATCAGTCAAAATAAAATCATTATTTGCC-- |
| recombinant DNA reagent | Synthesized putative ancestral S-domain surrounded by attL1 and attL2 sequences: SRKa*^MA^* | Thermo Fisher Scientific |  | >SRKa*^MA^*  CAAATAATGATTTTATTTTGACTGATAGTGACCTGTTCGTTGCAACAAATTGATGAGCAATGCTTTTTTATAATGCCAACTTTGTACAAAAAAGCAGGCTAGATGAGAGGTGCAGTACCAAATTACCACCATTCTCACAACTTTTTCTTCTTCCTGTTTGTTGTCTCAGTTCTGTTTCGTCCTGCGTTCTCGATCTTTGCCAATACTTTGTCGTCTACGGAATCTCTGACAATCGCAAGCAACCGAACCATCGTCTCTCTCGGTGATGATTTCGAACTTGGTTTCTTCAGACCCGCTGCAAGTCTTCGAGAGGGTGATCGTTGGTATCTCGGGATTTGGTACAAGACAATCTCGGTAAGAACCTATGTATGGGTTGCCAACAGAGATCATCCTATCTCCAGTTCTGATGGAACCCTCAAAATTTCCGGAATTAACCTTGTCCTCCTCAATCAATCTAACATCACTGTCTGGTCGACGAATCTGACTGGTGCTGTGAGATCTCCAGTGGTAGCAGAGCTTCTCCCTAATGGCAATTTCGTGCTTAGAGACTCCAAAACCAACGGGCAAGATGGATTTTTGTGGCAGAGCTTTGATTACCCGACAGATACTTTACTCCCGCATATGAAATTGGGTTTGGATCTCAAAACAGGAAACAACAGATTTCTTACATCCTGGAAAAACTCATATGATCCGTCAAGCGGGTATCTTTCGTACAAACTCGAAATGCAAGGGTTGCCTGAGTTTCTTATGTGGAGGAGCGGAGGGCCAGTGTTCCGGAGTGGTCCGTGGGATGGAATCCGGTTTAGTGGCATACCAGAGATGCAAAGATGGAAGTTCGTTAACATTGTTTACAATTTCACGGAGAACAAAGAGGATGTCGCCTTCACTTATAGAGTTACCACCCCCAATGTCTACGCGAAATTGACGATGAGATTCGACGGGTTTTTAGAACTATCCACATGGGATCCGGAAATGTTGGAATGGAACGTGTTCTGGGTTTCATCGACAGCCGATTGTGATATTTACATGGGGTGTACTCCTTATAGCTTCTGTGACACGAACACAACGCCAAAGTGTAACTGTATCAAAGGATTTGAGCCGAGGAACCCTCAGGGAGGAGCATTGGATAACACATCCACTGAGTGTGTAAGGAAGACGCAGTTAAATTGCAATGGAGATGGATTTTTCTGGCTGAGGAATATGAAGCTGCCGGACACATCGGGTGCTATTGTGGACAAGAGGATTGGGTTAAAGGAATGTGAGGAGAGGTGCATTGAGAATTGTAACTGTACTGCGTTTGCTAATACGAATATCCAAGATGGTGGGTCGGGTTGTGTGCTTTGGACTCGCGAGCTCGCAGATATTCGGAGATATGTCGATGCGGGTCAAGATCTTTATGTCAGATTGGCTGCTGTTGATCTCGGTTAGCTCTATCCCGTAGAATTATTACACGTAATGTATCCTGAAAATTCAGGGATCCACCCAAATTACCCAGCTTTCTTGTACAAAGTTGGCATTATAAGAAAGCATTGCTTATCAATTTGTTGCAACGAACAGGTCACTATCAGTCAAAATAAAATCATTATTTGCC-- |
| recombinant DNA reagent | Synthesized putative ancestral S-domain surrounded by attL1 and attL2 sequences: SRKa*^MG^* | Thermo Fisher Scientific |  | >SRKa*^MG^*  CAAATAATGATTTTATTTTGACTGATAGTGACCTGTTCGTTGCAACAAATTGATGAGCAATGCTTTTTTATAATGCCAACTTTGTACAAAAAAGCAGGCTAGATGAGAGGTGCAGTACCAAATTACCACCATTCTCACAACTTTTTCTTCTTCCTGTTTGTTGTCTCAGTTCTGTTTCGTCCTGCGTTCTCGATCTTTGCCAATACTTTGTCGTCTACGGAATCTCTGACAATCGCAAGCAACCGAACCATCGTCTCTCTCGGTGATGATTTCGAACTTGGTTTCTTCAGACCCGCTGCAAGTCTTCGAGAGGGTGATCGTTGGTATCTCGGGATTTGGTACAAGACAATCTCGGTAAGAACCTATGTATGGGTTGCCAACAGAGATCATCCTATCTCCAGTTCTGATGGAACCCTCAAAATTTCCGGAATTAACCTTGTCCTCCTCAATCAATCTAACATCACTGTCTGGTCGACGAATCTGACTGGTGCTGTGAGATCTCCAGTGGTAGCAGAGCTTCTCCCTAATGGCAATTTCGTGCTTAGAGACTCCAAAACCAACGGGCAAGATGGATTTTTGTGGCAGAGCTTTGATTACCCGACAGATACTTTACTCCCGCATATGAAATTGGGTTTGGATCTCAAAACAGGAAACAACAGATTTCTTACATCCTGGAAAAACTCATATGATCCGTCAAGCGGGTATCTTTCGTACAAACTCGAAATGCAAGGGTTGCCTGAGTTTCTTATGTGGAGGAGCGGAGGGCCAGTGTTCCGGAGTGGTCCGTGGGATGGAATCCGGTTTAGTGGCATACCAGAGATGCAAAGATGGAAGTTCGTTAACATTGTTTACAATTTCACGGAGAACAAAGAGGATGTCGCCTTCACTTATAGAGTTACCACCCCCAATGTCTACGCGAAATTGACGATGAGATTCGACGGGTTTTTAGAACTATCCACATGGGATCCGGAAATGTTGGAATGGAACGTGTTCTGGGTTTCATCGACAGGCGATTGTGATATTTACATGGGGTGTACTCCTTATAGCTTCTGTGACACGAACACAACGCCAAAGTGTAACTGTATCAAAGGATTTGAGCCGAGGAACCCTCAGGGAGGAGCATTGGATAACACATCCACTGAGTGTGTAAGGAAGACGCAGTTAAATTGCAATGGAGATGGATTTTTCTGGCTGAGGAATATGAAGCTGCCGGACACATCGGGTGCTATTGTGGACAAGAGGATTGGGTTAAAGGAATGTGAGGAGAGGTGCATTGAGAATTGTAACTGTACTGCGTTTGCTAATACGAATATCCAAGATGGTGGGTCGGGTTGTGTGCTTTGGACTCGCGAGCTCGCAGATATTCGGAGATATGTCGATGCGGGTCAAGATCTTTATGTCAGATTGGCTGCTGTTGATCTCGGTTAGCTCTATCCCGTAGAATTATTACACGTAATGTATCCTGAAAATTCAGGGATCCACCCAAATTACCCAGCTTTCTTGTACAAAGTTGGCATTATAAGAAAGCATTGCTTATCAATTTGTTGCAACGAACAGGTCACTATCAGTCAAAATAAAATCATTATTTGCC-- |
| sequence-based reagent | Primer attB1.-1940.-F | Thermo Fisher Scientific |  | 5’-GGGGACAAGTTTGTACAAAAAAGCAGGCTAACCCTGGCTTACTGACTTG-3’ |
| sequence-based reagent | Primer attB2.4555.R | Thermo Fisher Scientific |  | 5’-GGGGACCACTTTGTACAAGAAAGCTGGGTAATCGCCCGGTTATTGCCTG-3’ |
| sequence-based reagent | Primer attB1.-1950.F | Thermo Fisher Scientific |  | 5’-GGGGACAAGTTTGTACAAAAAAGCAGGCTAGGTTAGTCCATAGCCCTTG-3’ |
| sequence-based reagent | Primer attB2.4555.R | Thermo Fisher Scientific |  | 5’-GGGGACCACTTTGTACAAGAAAGCTGGGTAATCGCCCGGTTATTGCCTG-3’ |
| sequence-based reagent | Primer attB1.-1940.F | Thermo Fisher Scientific |  | 5’-GGGGACAAGTTTGTACAAAAAAGCAGGCTAACCCTGGCTTACTGACTTG-3’ |
| sequence-based reagent | Primer attB2.-1.R | Thermo Fisher Scientific |  | 5’-GGGGACCACTTTGTACAAGAAAGCTGGGTCTCTCTCTCTACCACTGTGC-3’ |
| sequence-based reagent | Primer attB1.-1950.F | Thermo Fisher Scientific |  | 5’-GGGGACAAGTTTGTACAAAAAAGCAGGCTAGGTTAGTCCATAGCCCTTG-3’ |
| sequence-based reagent | Primer attB2.-3.R | Thermo Fisher Scientific |  | 5’-GGGGACCACTTTGTACAAGAAAGCTGGGTCTCTCTCTACCACTGTGCTC-3’ |
| sequence-based reagent | Primer attB4.-1940.F | Thermo Fisher Scientific |  | 5’-GGGGACAACTTTGTATAGAAAAGTTGAACCCTGGCTTACTGACTTG-3’ |
| sequence-based reagent | Primer attB1r.-1.R | Thermo Fisher Scientific |  | 5’-GGGGACTGCTTTTTTGTACAAACTTGCTCTCTCTCTACCACTGTGC-3’ |
| sequence-based reagent | Primer attB2r.1393.F | Thermo Fisher Scientific |  | 5’-GGGGACAGCTTTCTTGTACAAAGTGGCAACTTCCGGTTCCATATCC-3’ |
| sequence-based reagent | Primer attB3.4555.R | Thermo Fisher Scientific |  | 5’-GGGGACAACTTTGTATAATAAAGTTGAATCGCCCGGTTATTGCCTG-3’ |
| sequence-based reagent | Primer attB4.-1950.F | Thermo Fisher Scientific |  | 5’-GGGGACAACTTTGTATAGAAAAGTTGAGGTTAGTCCATAGCCCTTG-3’ |
| sequence-based reagent | Primer attB1r.-3.R | Thermo Fisher Scientific |  | 5’-GGGGACTGCTTTTTTGTACAAACTTGCTCTCTCTACCACTGTGCTC-3’ |
| sequence-based reagent | Primer attB2r.1393.F | Thermo Fisher Scientific |  | 5’-GGGGACAGCTTTCTTGTACAAAGTGGCAACTTCCGGTTCCATATCC-3’ |
| sequence-based reagent | Primer attB3.4555.R | Thermo Fisher Scientific |  | 5’-GGGGACAACTTTGTATAATAAAGTTGAATCGCCCGGTTATTGCCTG-3’ |
| sequence-based reagent | Gateway™ pDONR™221 Vector | Thermo Fisher Scientific | 12536017 |  |
| sequence-based reagent | pK7WG | VIB-UGent Center for Plant Systems Biology | Vector ID: 4.38 |  |
| sequence-based reagent | pKGWFS7.0 | VIB-UGent Center for Plant Systems Biology | Vector ID: 1.19 |  |
| sequence-based reagent | Random Hexamer Primer | Thermo Fisher Scientific | SO142 |  |
| sequence-based reagent | Primers for cDNA amplification of *SRK* fragments | Thermo Fisher Scientific |  | For: AGGAATGTGAGGAGAGGTGC  Rev: TCCTACTGTTGTTGTTGCCC |
| sequence-based reagent | Primers for cDNA amplification of *Ubiquitin* housekeeping gene | Thermo Fisher Scientific  PMID: 17412590 |  | For: CTGAGCCGGACAGTCCTCTTAACTG  Rev: CGGCGAGGCGTGTATACATTTGTG |
| peptide, recombinant protein | primeSTAR® DNA polymerase | Takara | Cat. #R045A |  |
| peptide, recombinant protein | Gateway™ BP Clonase™ II Enzyme mix | Thermo Fisher Scientific | 11789020 |  |
| peptide, recombinant protein | Gateway™ LR Clonase™ II Enzyme Mix | Thermo Fisher Scientific | 11791-020 |  |
| peptide, recombinant protein | RevertAid Reverse Transcriptase | Thermo Fisher Scientific | EP0442 |  |
| peptide, recombinant protein | Riboblock RNase inhibitor | Thermo Fisher Scientific | EO0382 |  |
| commercial assay or kit | MultiSite Gateway® Three-Fragment Vector Construction Kit | Thermo Fisher Scientific | 12537-023 |  |
| commercial assay or kit | nucleospin RNA Plus Extraction kit | Macherey Nagel | 740984 |  |
| chemical compound, drug | formaldehyde | Merck | F8775 |  |
| chemical compound, drug | acetic acid | Merck | 338826 |  |
| chemical compound, drug | aniline blue | Merck | 128-M |  |
| software, algorithm | MACSE v1.2 | PMID: [21949676](https://www.ncbi.nlm.nih.gov/pubmed/21949676/) |  |  |
| software, algorithm | PHYML 3.0 | PMID: [20525638](https://www.ncbi.nlm.nih.gov/pubmed/20525638/)  RRID:SCR_014629 |  |  |
| software, algorithm | MrBayes 3.2.4 | PMID: [12912839](https://www.ncbi.nlm.nih.gov/pubmed/12912839/)  RRID:SCR_012067 |  |  |
| software, algorithm | jModelTest 2.1.10 | PMID: [22847109](https://www.ncbi.nlm.nih.gov/pubmed/22847109/)  RRID:SCR_015244 |  |  |
| software, algorithm | PAML 4.8 package (codeml program) | PMID: [17483113](https://www.ncbi.nlm.nih.gov/pubmed/17483113/)  RRID:SCR_014932 |  |  |
| software, algorithm | MODELLER | PMID: 8254673  RRID:SCR_008395 |  |  |
| software, algorithm | GalaxyRefineComplex web service | PMID: [27535582](https://www.ncbi.nlm.nih.gov/pubmed/27535582) |  | http://galaxy.seoklab.org/refinecomplex |
| software, algorithm | PyMol molecular graphics system, version 1.7.2.1 | Schrodinger LLC  RRID:SCR_006054 |  |  |
