# Supplementary material for: Asymmetrical diversification of the receptor-ligand interaction controlling self-incompatibility in Arabidopsis
Source: eLife. 2019 Nov 25;8:e50253. doi: 10.7554/eLife.50253 (PMC6908432; doi:10.7554/eLife.50253)
Supplement: Transparent reporting form [file elife-50253-transrepform.docx]

***eLife’s* transparent reporting form**

We encourage authors to provide detailed information *within their submission* to facilitate the interpretation and replication of experiments. Authors can upload supporting documentation to indicate the use of appropriate reporting guidelines for health-related research (see [EQUATOR Network](http://www.equator-network.org/%20)), life science research (see the [BioSharing Information Resource](https://biosharing.org/)), or the [ARRIVE guidelines](http://www.plosbiology.org/article/info:doi/10.1371/journal.pbio.1000412) for reporting work involving animal research. Where applicable, authors should refer to any relevant reporting standards documents in this form.

If you have any questions, please consult our Journal Policies and/or contact us: [editorial@elifesciences.org](mailto:editorial@elifesciences.org).

**Sample-size estimation**

- You should state whether an appropriate sample size was computed when the study was being designed
- You should state the statistical method of sample size computation and any required assumptions
- If no explicit power analysis was used, you should describe how you decided what sample (replicate) size (number) to use

Please outline where this information can be found within the submission (e.g., sections or figure legends), or explain why this information doesn’t apply to your submission:

- No sample size was explicitly computed when the study was being designed.

- However, the study is focused on *n*=2 allelic lines that were chosen based on their close phylogenetic proximity (S3 and S28). For each allelic line, successful transfer of the SI specificity phenotype from *Arabidopsis halleri* into *A. thaliana* was verified by n=2 replicate transgenic lines. The ancestral SI specificity was determined based on an average of n=6 transgenic lines. These numbers are larger than most published studies in the field, where a single transgenic line is typically characterized in details.

**Replicates**

- You should report how often each experiment was performed
- You should include a definition of biological versus technical replication
- The data obtained should be provided and sufficient information should be provided to indicate the number of independent biological and/or technical replicates
- If you encountered any outliers, you should describe how these were handled
- Criteria for exclusion/inclusion of data should be clearly stated
- High-throughput sequence data should be uploaded before submission, with a private link for reviewers provided (these are available from both GEO and ArrayExpress)

Please outline where this information can be found within the submission (e.g., sections or figure legends), or explain why this information doesn’t apply to your submission:

- In our **transgenic experiments**, biological replicates are the different transformed *A. thaliana* lines used to characterize the SI response. *n*=2 replicate lines were used to verify successful transfer of the SI specificity phenotype from *Arabidopsis halleri* into *A. thaliana* (Figure 2). An average of *n*=6 replicate lines were used to evaluate SI specificity of the reconstructed ancestors (Figure 3 and S7). Technical replicates are the number of pollinated pistils to quantify the SI response by counting the number of germinating pollen tubes (n=6.5 on average; indicated on top of the bars Figures 2, 3 and S7).

- For the **RNA quantification** experiment presented on Figure S6, biological replicates correspond to RNA isolated from floral buds of three separate individuals for each line (separate extraction and reverse transcription). Each biological replicate was then subjected to three qPCR experiments (technical replicates).

These information can be found on the boxplots directly and in the legend of Figure S6.

**Statistical reporting**

- Statistical analysis methods should be described and justified
- Raw data should be presented in figures whenever informative to do so (typically when N per group is less than 10)
- For each experiment, you should identify the statistical tests used, exact values of N, definitions of center, methods of multiple test correction, and dispersion and precision measures (e.g., mean, median, SD, SEM, confidence intervals; and, for the major substantive results, a measure of effect size (e.g., Pearson's r, Cohen's d)
- Report exact p-values wherever possible alongside the summary statistics and 95% confidence intervals. These should be reported for all key questions and not only when the p-value is less than 0.05.

Please outline where this information can be found within the submission (e.g., sections or figure legends), or explain why this information doesn’t apply to your submission:

- Ancestral state reconstruction was conducted by taking uncertainty of the phylogenetic inference into account. We compared the likelihood of a series of codon-based models and the best-fitting model was determined by Akaike information criterion. Details of the phylogenetic analysis can be found at the beginning of the Materials and Methods section.

(For large datasets, or papers with a very large number of statistical tests, you may upload a single table file with tests, Ns, etc., with reference to sections in the manuscript.)

**Group allocation**

- Indicate how samples were allocated into experimental groups (in the case of clinical studies, please specify allocation to treatment method); if randomization was used, please also state if restricted randomization was applied
- Indicate if masking was used during group allocation, data collection and/or data analysis

Please outline where this information can be found within the submission (e.g., sections or figure legends), or explain why this information doesn’t apply to your submission:

The count of pollen tubes was made blindly in a randomized manner.

**Additional data files (“source data”)**

- We encourage you to upload relevant additional data files, such as numerical data that are represented as a graph in a figure, or as a summary table
- Where provided, these should be in the most useful format, and they can be uploaded as “Source data” files linked to a main figure or table
- Include model definition files including the full list of parameters used
- Include code used for data analysis (e.g., R, MatLab)
- Avoid stating that data files are “available upon request”

Please indicate the figures or tables for which source data files have been provided:

Figure 3: data file_fig3

Figure S7: data file_figS7
